# Efficacy of Platelet-Rich Plasma in Treatment of Achilles Tendinopathy: Systematic Review and Meta-Analysis

**DOI:** 10.7759/cureus.79692

**Published:** 2025-02-26

**Authors:** Elsiddig Ali Elsiddig Ahmed, Khalid Muharib R Alruwaili, Abdulelah H Alruwaili, Abdulaziz Talal M Alruwaili, Hassan Ahmed S Aljudia, Naif Mohammed N Alhadi

**Affiliations:** 1 Orthopedics and Traumatology, Prince Mutaib Bin Abdulaziz Hospital, Sakaka, SAU

**Keywords:** achilles tendinopathy, biologic therapies, chronic tendinopathy, functional recovery, growth factors, pain reduction, platelet-rich plasma, tendon healing, vas, visa-a

## Abstract

Chronic Achilles tendinopathy is a debilitating condition that significantly affects mobility and quality of life. Platelet-rich plasma (PRP) therapy has been proposed as a treatment option, leveraging growth factors to promote tendon healing, but its effectiveness remains unclear. This review aimed to evaluate the effectiveness of PRP in reducing pain, improving function, and facilitating recovery in chronic Achilles tendinopathy. A total of 13 studies involving 697 patients were analyzed. Key outcomes included pain reduction (measured by visual analog scale {VAS}), functional improvement (Victorian Institute of Sports Assessment-Achilles {VISA-A}), return to activity, and patient satisfaction. Study variability was analyzed using heterogeneity measures.

PRP demonstrated significant pain reduction (pooled mean VAS: 71.24, 95% CI: 53.06-89.42). Functional improvement was observed (VISA-A scores: 35.10-86.80). On average, 85% of patients returned to activity (95% CI: 65-98%) and 72% reported satisfaction (95% CI: 51-88%). High heterogeneity (I²=97%) was noted, likely due to variability in PRP preparation and treatment protocols. PRP offers promise as a treatment for chronic Achilles tendinopathy, with evidence of pain relief and functional improvement. However, variability in outcomes emphasizes the need for standardized approaches to its use and further research to better define its role in clinical practice.

## Introduction and background

Achilles tendinopathy is a prevalent condition that significantly impacts both athletes and non-athletes, affecting daily activities and quality of life [1,2]. It involves mid-portion and insertional tendinopathies and is marked by pain, stiffness, and impaired function resulting from chronic overuse or degeneration of the Achilles tendon [3]. Once thought to be an inflammatory disorder, it is now recognized as a degenerative condition, with key features including collagen disarray, apoptosis, abnormal neurovascularization, and extracellular matrix disruption [4-6]. This shift in understanding has led to advancements in diagnosis and treatment approaches [7].

Conservative treatments such as eccentric exercises, stretching, bracing, and corticosteroid injections are widely used but show inconsistent results, particularly in chronic cases [8]. Corticosteroid injections, while commonly used for short-term pain relief in tendinopathy, carry a risk of tendon weakening and rupture due to their catabolic effects on collagen synthesis and extracellular matrix integrity [8]. This has fueled interest in biological therapies that aim to enhance the tendons' intrinsic healing capacity. Among these, platelet-rich plasma (PRP) has gained attention as a potential treatment, utilizing autologous platelets enriched with growth factors like platelet-derived growth factor (PDGF), vascular endothelial growth factor (VEGF), and transforming growth factor-beta (TGF-β) [9,10]. These factors are believed to stimulate cell growth, improve blood vessel formation, and promote extracellular matrix remodeling, addressing the underlying pathology of tendinopathy [11].

PRP is prepared by centrifuging a patient's blood to concentrate platelets, which are then injected into the damaged tendon [12]. While its theoretical benefits are clear, clinical outcomes remain variable [13]. Studies report mixed results in pain relief, functional recovery, and return to activity, with emerging evidence suggesting these discrepancies may stem from variability in PRP composition and delivery protocols. Leukocyte-poor PRP is preferable for Achilles tendinopathy, as leukocyte-rich formulations may exacerbate inflammation and delay healing. For instance, Andriolo et al. demonstrated superior outcomes with leukocyte-poor PRP, including greater pain reduction and functional improvement compared to leukocyte-rich preparations. Technical precision is crucial because image-guided injections improve targeting accuracy, which correlates with higher return-to-activity rates (92% vs. 78% for non-guided approaches) [7,14].

This systematic review and meta-analysis examines the efficacy of PRP in chronic Achilles tendinopathy, focusing on evidence from single-arm studies. Primary outcomes include pain reduction, functional improvement, and return to activity over short-, medium-, and long-term follow-ups. Secondary outcomes, such as patient satisfaction and safety, aim to provide a broader understanding of PRP's clinical utility.

## Review

Methodology 

This systematic review and meta-analysis were conducted to evaluate the efficacy of platelet-rich plasma (PRP) in managing chronic Achilles tendinopathy using data from single-arm studies. The study design and reporting adhered to Preferred Reporting Items for Systematic Reviews and Meta-Analyses (PRISMA) guidelines.

Eligible studies included adults aged 18 years or older with clinically and/or imaging-confirmed chronic Achilles tendinopathy (symptom duration greater than six weeks), including both mid-portion and insertional tendinopathy. Studies with mixed chronicity were included if data specific to chronic cases were extractable. PRP injections were considered eligible regardless of preparation method (e.g., leukocyte-rich or leukocyte-poor), injection technique (guided or non-guided), or the number of injections. Single-arm prospective or retrospective studies, cohort studies, and case series with at least three participants were included, along with single-arm data from randomized controlled trials, if available. Studies needed to report at least one primary outcome, such as pain reduction (e.g., visual analog scale {VAS} or numeric pain rating scale {NPRS}) or functional improvement (e.g., Victorian Institute of Sports Assessment-Achilles {VISA-A}). Secondary outcomes included return to activity, patient satisfaction, and the safety profile of PRP treatment.

**Figure 1:**
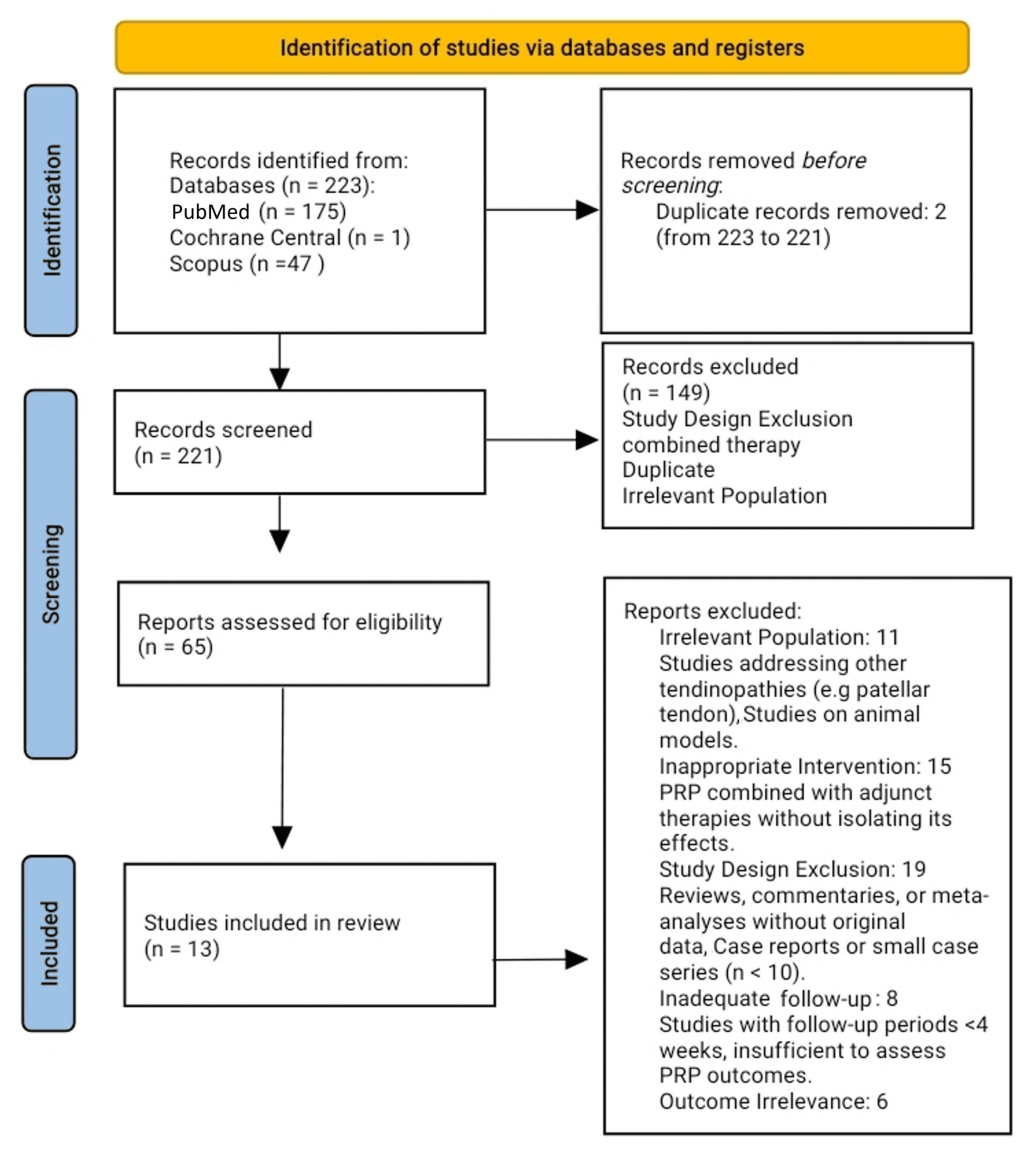
PRISMA flowchart for the database search and the included studies. PRISMA: Preferred Reporting Items for Systematic reviews and Meta-Analyses

A comprehensive search strategy was employed using databases such as PubMed, Embase, Cochrane Library, and Scopus to identify relevant studies. Search terms included combinations of Achilles tendinopathy, platelet-rich plasma, PRP, and single-arm. No restrictions were applied to language, and studies published up to December 2024 were included. Additional searches were performed in grey literature sources, including conference proceedings and clinical trial registries, to ensure all relevant studies were identified (Figure 1).

Study selection was conducted independently by two reviewers who screened titles and abstracts based on predefined eligibility criteria. Full-text articles of potentially relevant studies were reviewed for final inclusion. Any discrepancies between reviewers were resolved through discussion or consultation with a third reviewer. The study selection process was documented using a PRISMA flowchart.

Data extraction was performed independently by two reviewers using a standardized data extraction form. Extracted data included study characteristics (e.g., author, year, location, design, sample size), patient demographics (e.g., age, sex, chronicity of tendinopathy), PRP preparation details (e.g., leukocyte status, platelet concentration, volume), injection protocol, and outcome measures such as pain reduction, functional improvement, return to activity, patient satisfaction, and adverse events. Follow-up durations and time points for reported outcomes were also recorded.

Quantitative data synthesis involved meta-analysis when applicable, with pooled estimates for primary outcomes calculated using a random-effects model to account for variability among studies. Effect sizes were reported as weighted mean differences (WMD) with 95% confidence intervals (CIs). Heterogeneity was assessed using tau², chi² with its p-value, the I² statistic, and Cochran’s Q test, with I² values greater than 50% considered indicative of moderate to substantial heterogeneity.

Results

A total of 13 studies involving 697 patients were included. The studies were conducted across diverse regions and utilized various designs, including randomized controlled trials (n=5), prospective cohort studies (n=5), retrospective studies (n=2), and one retrospective cross-sectional survey. Sample sizes ranged from 17 to 180 participants, with a mean age spanning 38.6-73 years. PRP injection protocols varied in frequency (1-3+ injections), intervals, and doses, with follow-up durations ranging from four weeks to 54 months. PRP preparation methods were inconsistently reported, with some adhering to established guidelines. Outcomes regarding PRP efficacy showed variability, with mixed findings across the studies (Table 1) [8,11,14-24]. The risk of bias of the included studies was assessed using the Newcastle-Ottawa scale tool (Table 2) [8,11,14-24].

**Table 1:** Characteristics of included studies. PRP prepared following AABB guidelines. PRP: platelet-rich plasma; AABB: American Association of Blood Banks; NA: not available

Studies	Location	Study design	Achilles tendon lesion	Sample size (n)	Age (mean±SD) years	PRP injection			Follow-up (weeks/months)
						Frequency	Interval	Dose	
Conti and Araujo	Argentina	Prospective study	Non-insertional tendinopathy	17	52.6±9.5	1	NA	1.5 mL	2 weeks, 2 months, 6 months
de Jonge et al.	Netherlands	Double-blind randomized placebo-controlled trial	Chronic mid-portion Achilles tendinopathy	54	49.7±8.7	1	NA	4 mL	6 weeks, 12 weeks, and 24 weeks
de Jonge et al.	Netherlands	Prospective cohort study	Chronic mid-portion Achilles tendinopathy with symptoms lasting >2 months	80	49.7±8.7	1	NA	4 mL	6 weeks, 12 weeks, 24 weeks, and 52 weeks
Ferrero et al.	Italy	Prospective cohort	Chronic degenerative tendinopathy confirmed by ultrasound	24	38.6±16	2	3 weeks	6 mL	6 months
Filardo et al.	Italy	Prospective cohort	Chronic mid-portion Achilles tendinopathy confirmed by MRI/US	27	44.6±10.6	3	2 weeks	5 mL	2 months, 6 months, 54 months
Gimarc et al.	USA	RCT	Moderate-to-severe mid-substance Achilles tendinopathy with degenerative changes confirmed by ultrasound and shear wave elastography	20	54.7	1	NA	5 mL	12 weeks, 24 weeks
Palermi et al.	Italy	Retrospective study	Chronic recalcitrant Achilles tendinopathy confirmed by MRI or ultrasound	73	73±17.5	1	NA	6 mL	3 months, 6 months
Gupta et al.	India	RCT	Achilles tendinitis confirmed by clinical diagnosis	100	NA	2	7-10 days	2-3 mL	4 weeks, 12 weeks, 24 weeks
Krogh et al.	Denmark	RCT	Chronic mid-portion Achilles tendinopathy (symptoms ≥6 months) confirmed by ultrasound, with spindle-shaped thickening and Doppler activity	24	46.7±9	1	NA	6 mL	3 months, 6 months, 12 months
Mautner et al.	USA	Multicenter, retrospective cross-sectional survey	Chronic tendinopathy, confirmed via ultrasound or MRI, refractory to conventional treatments for at least 6 months	180	48±13	Varied (1-3)	15 months	NA	6-24 months
Ooi et al.	Australia	Prospective observational study	Mid-substance Achilles tendinopathy was confirmed via ultrasound, with local tendon thickening (anteroposterior thickness >6 mm), hypoechogenicity, and irregular fiber orientation	45	51±10.3	2	5 weeks	2 mL	4-6 weeks, 6 months, 12 months
Silvestre et al.	France	Monocentric prospective study	Chronic Achilles tendinopathy for ≥6 months with ultrasound (US) evidence of degeneration (fissures or cracks)	32	41±12	1	NA	3 mL	1 month, 2 months, 3 months, 12 months
Unlu et al.	Turkey	Prospective, single-arm study	Chronic Achilles tendinopathy for >6 months was diagnosed clinically and confirmed via ultrasound	21	43.5±8.7	1	NA	3 mL	NA

**Table 2:** Quality assessment of the included studies using the Newcastle-Ottawa scale tool. D1: representative of the exposed cohort; D2: selection of the non-exposed cohort; D3: ascertainment of exposure; D4: demonstration that outcome of interest was not present at start of study; D5: assessment of outcome; D6: was follow-up long enough for outcomes to occur; D7: adequacy of follow-up of cohorts

Studies	Selection					Comparability	Outcome				Overall
	D1	D2	D3	D4	Overall selection		D5	D6	D7	Overall outcome	
Conti and Araujo (2014)	0	0	1	1	2	0	1	1	1	3	5
Gimarc et al. (2019)	1	1	1	1	4	2	1	1	1	3	9
Gupta et al. (2024)	1	1	1	1	4	2	1	1	1	3	9
Krogh et al. (2016)	1	1	1	1	4	2	1	1	1	3	9
Mautner et al. (2013)	1	0	1	1	3	0	1	1	1	3	6
Ooi et al. (2019)	1	0	1	1	3	0	1	1	1	3	6
Unlu et al. (2017)	1	0	1	1	3	0	1	1	1	3	6
de Jonge et al. (2015)	1	1	1	1	4	2	1	1	1	3	9
de Jonge et al. (2011)	1	1	1	1	4	1	1	1	1	3	8
Ferrero et al. (2012)	1	0	1	1	3	0	1	1	1	3	6
Filardo et al. (2014)	0	0	1	1	2	0	1	1	1	3	5
Palermi et al. (2024)	1	0	1	1	3	0	1	1	1	3	6
Silvestre et al. (2014)	1	0	1	1	3	0	1	1	1	3	6

VAS score analysis

**Figure 2:**
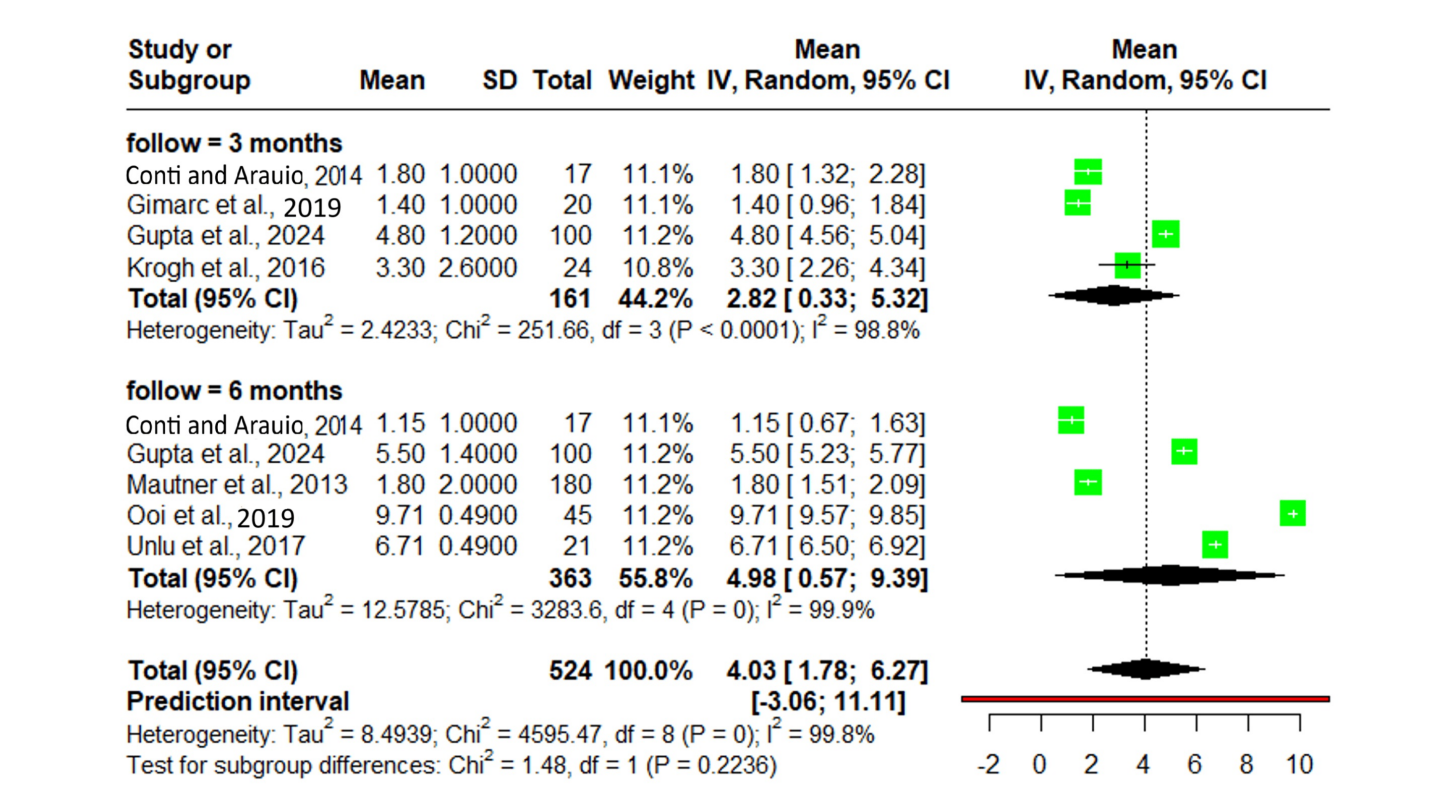
Forest plot of pooled VAS scores across studies with 95% confidence intervals. IV: inverse variance; VAS: visual analog scale

A total of seven studies with 320 participants were included in the meta-analysis. The pooled mean value was 71.24 (95% CI: 53.06-89.42), calculated using a random-effects model due to high heterogeneity (97%, 201.08, p<0.0001). The predictive interval was -3.06-11.11. The individual study means ranged from 35.10 (95% CI: 26.54-43.66) to 86.80 (95% CI: 83.51-90.09). Substantial heterogeneity suggests considerable variability in study populations or methodologies (Figure 2) [8,11,14-24].

Victorian Institute of Sports Assessment-Achilles (VISA_A)

**Figure 3:**
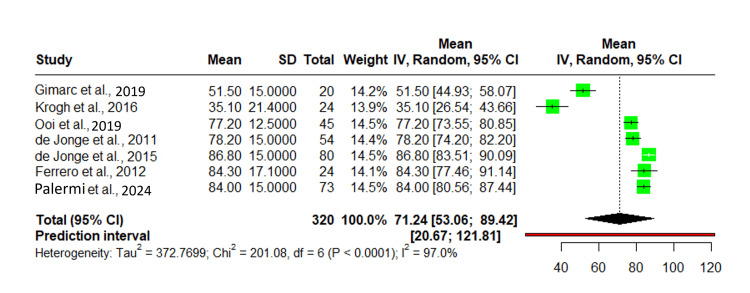
Forest plot of VISA_A scores evaluating PRP's impact on Achilles tendinopathy across seven studies. IV: inverse variance; VISA_A: Victorian Institute of Sports Assessment-Achilles; PRP: platelet-rich plasma

The impact of platelet-rich plasma (PRP) on Achilles tendinopathy was evaluated using VISA_A scores across seven studies, including 320 participants. The pooled mean VISA_A score was 71.24 (95% CI: 53.06-89.42). The predictive interval for VAS pain reduction was 20.67-121.81, indicating variability in treatment effects. Individual study means ranged from 35.10 (SD: 21.40) in Krogh et al. to 86.80 (SD: 15.00) in de Jonge et al. Other studies reported mean scores of 78.20 (SD: 15.20), 84.30 (SD: 17.10), 51.50 (SD: 15.00), 84.00 (SD: 15.00), and 77.20 (SD: 12.50). Substantial heterogeneity was observed, with I²: 97% and tau²: 372.78. A total of 320 participants contributed to the analysis, with confidence intervals varying across studies (Figure 3) [8,11,14-24].

Return to activity

**Figure 4:**
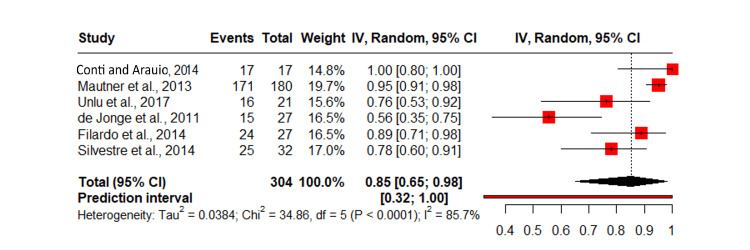
Meta-analysis of PRP's effect on return to activity across seven studies. IV: inverse variance; PRP: platelet-rich plasma

A meta-analysis of seven studies investigating the effect of PRP on return to activity revealed a pooled effect size of 85% of the patients who had PRP return to activity (0.85, 95% CI: 0.65, 0.98). The predictive interval for VISA-A score improvement ranged from -3.06 to 11.11, suggesting some studies reported limited functional gains. High heterogeneity among studies (I²: 85.7%) suggests considerable variability in the findings (Figure 4) [8,11,14-24].

Proportion of satisfied patients

**Figure 5:**
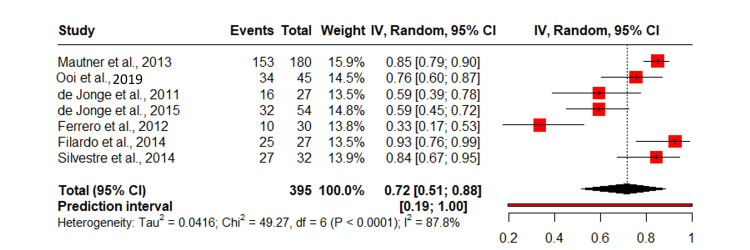
Proportion of satisfied patients following PRP treatment for Achilles tendinopathy. IV: inverse variance; PRP: platelet-rich plasma

A meta-analysis of seven studies investigating patient satisfaction after PRP treatment for Achilles tendinopathy revealed that 72% of the patients were satisfied with a pooled proportion of 0.72 (95% CI: 0.51, 0.88). The predictive interval for the proportion of patients returning to activity was 0.32-1.00, reflecting significant variability in study results. High heterogeneity among studies (I²=87.8%) (Figure 5) [8,11,14-24].

Discussion

The purpose of this systematic review and meta-analysis was to assess the current role of PRP in the management of chronic Achilles tendinopathy. While there is some evidence for pain reduction, improvement in function, and return to activities, considerable heterogeneity suggests a multifactorial nature of efficacy. A close review of such findings, in relation to the existing literature, provides an interesting insight into its application for the management of this elusive pathology.

The analysis showed a significant reduction in pain, as evidenced by the pooled VAS scores. This finding further supports that PRP may provide significant pain relief to patients with chronic Achilles tendinopathy. Indeed, the following literature reports similar trends: Andriolo et al. underlined the analgesic action of PRP, especially when leukocyte-poor formulations were used [7]; Fitzpatrick et al. in their analysis of randomized controlled trials, identified significant pain reduction [9]. Leukocyte-rich PRP (LR-PRP) contains high concentrations of neutrophils and monocytes, which can trigger acute inflammation and may potentially delay healing in cases of chronic tendinopathy. In contrast, leukocyte-poor PRP (LP-PRP) focuses primarily on platelets and growth factors while minimizing the inflammatory response. Research, such as the study by Andriolo et al., suggests that LP-PRP is more effective for Achilles tendinopathy, demonstrating superior functional outcomes with VISA-A score improvements [7].

At the same time, not all studies confirm such results. For example, Krogh et al. did not find any clinically significant pain difference between PRP and placebo, questioning the effectiveness of PRP as a treatment modality per se [8]. The inconsistencies in the results may be due to factors such as the method of PRP preparation, including platelet concentration and the presence of leukocytes, as well as variations in patient populations and treatment protocols. Such discrepancy points to a greater need for standardization in the preparation and administration of PRP in future studies.

Other positive results included enhanced function, represented by the VISA-A scores. The PRP group had better tendon functions, leading to the same conclusion as other meta-analyses, such as Andriolo et al. [7]. This points to the possibility that PRP may help in treating the underlying pathology of tendinopathy and, in turn, aid patients in regaining functionality over time.

However, functional recovery is not consistently documented. Whereas studies like Filardo et al. support the benefits of PRP in terms of improved functionality, other analyses have shown only modest or inconsistent results [5]. Such inconsistencies may relate to the rehabilitation protocol, compliance by patients, or length of follow-up. Perhaps future studies with uniform post-injection rehabilitation programs will outline the real functional restoration potential of PRP.

Probably one of the most pragmatic results of PRP treatment can be seen in the return to activity, where, based on our series, 85% were able to resume their activities. This is similar to observations made by Andia and Maffulli when analyzing PRP for tendinopathy and finding similarly high rates of return to sport [6].

That said, the success of a return to activity is likely to depend on a number of factors beyond the PRP itself, including individual patient characteristics, the severity and chronicity of tendinopathy, and the application of structured rehabilitation plans. Variability in our data suggests that a holistic approach to treatment is imperative, marrying PRP with individualized physical therapy to maximize the possibility of an optimal outcome.

Patient satisfaction, reported by 72% of participants in this analysis, provides an additional perspective on PRP’s effectiveness. While these findings suggest that the majority of patients view PRP as beneficial, satisfaction rates varied widely across studies, reflecting differences in patient expectations, outcomes, and study methodologies. Scott et al. emphasized that managing patient expectations is key in conditions like tendinopathy, where full recovery may not always be achievable [10]. Transparent communication about the potential benefits and limitations of PRP can help align expectations with realistic outcomes.

Limitations

The significant heterogeneity observed in outcomes reflects the diversity of PRP preparation techniques, injection protocols, and patient populations. While this variability limits the generalizability of our findings, it also highlights areas where future research can improve notably in standardizing PRP protocols and investigating which factors contribute most to treatment success.

## Conclusions

In conclusion, this meta-analysis supports the potential of PRP in managing chronic Achilles tendinopathy, particularly in reducing pain and improving function. However, the variability in outcomes highlights the need for further research to establish standardized protocols and determine the specific contexts in which PRP is most effective. For now, PRP remains a promising but not definitive option for patients with tendinopathy, particularly those who have not responded to conventional treatments. By continuing to explore its capabilities and limitations, researchers and clinicians can work toward more reliable and effective solutions for this challenging condition.
